# Identification and validation of a cigarette smoke-related five-gene signature as a prognostic biomarker in kidney renal clear cell carcinoma

**DOI:** 10.1038/s41598-022-06352-y

**Published:** 2022-02-09

**Authors:** Yefei Huang, Qinzhi Wang, Yu Tang, Zixuan Liu, Guixiang Sun, Zhaojun Lu, Yansu Chen

**Affiliations:** 1 Xuzhou Key Laboratory of Environment and Health, School of Public Health, Xuzhou Medical University, Xuzhou, 221004 Jiangsu Province China; 2 Second Clinical Medical College, Xuzhou Medical University, Xuzhou, 221004 Jiangsu Province China

**Keywords:** Tumour biomarkers, Renal cancer

## Abstract

Cigarette smoking greatly promotes the progression of kidney renal clear cell carcinoma (KIRC), however, the underlying molecular events has not been fully established. In this study, RCC cells were exposed to the tobacco specific nitrosamine 4-(methylnitrosamino)-1-(3-pyridyl)-1-butanone (NNK, nicotine-derived nitrosamine) for 120 days (40 passages), and then the soft agar colony formation, wound healing and transwell assays were used to explore characteristics of RCC cells. RNA-seq was used to explore differentially expressed genes. We found that NNK promoted RCC cell growth and migration in a dose-dependent manner, and RNA-seq explored 14 differentially expressed genes. In TCGA-KIRC cohort, Lasso regression and multivariate COX regression models screened and constructed a five-gene signature containing ANKRD1, CYB5A, ECHDC3, MT1E, and AKT1S1. This novel gene signature significantly associated with TNM stage, invasion depth, metastasis, and tumor grade. Moreover, when compared with individual genes, the gene signature contained a higher hazard ratio and therefore had a more powerful value for the prognosis of KIRC. A nomogram was also developed based on clinical features and the gene signature, which showed good application. Finally, AKT1S1, the most crucial component of the gene signature, was significantly induced after NNK exposure and its related AKT/mTOR signaling pathway was dramatically activated. Our findings supported that NNK exposure would promote the KIRC progression, and the novel cigarette smoke-related five-gene signature might serve as a highly efficient biomarker to identify progression of KIRC patients, AKT1S1 might play an important role in cigarette smoke exposure-induced KIRC progression.

## Introduction

Kidney and renal pelvis cancer is among the top ten most common cancers in the world, with 65,340 new cases and 14,970 deaths in 2018 in United States^1^. Renal cell carcinoma (RCC) accounts for ~ 85% of all renal malignancies, and kidney renal clear cell carcinoma (KIRC) arising from the proximal convoluted tubule is the most common and malignant histological subtype and responsible for most of deaths among all the subtypes of RCC^2^.

The link between cigarette smoking and RCC has been well-established. Active smoking is associated with histological RCC subtype, especially with KIRC^3,4^. The relative risk of KIRC is not only higher in smokers as compared to non-smokers, but also increased with the cumulative dose and duration of smoking^5,6^. Moreover, cigarette smoking has an ongoing effect on the progression of KIRC. Studies have validated that tobacco exposure is positively associated with aggressive clinical parameters of KIRC and decreases the cancer-specific survival and overall survival of KIRC patients^7,8^. However, the underlying molecular events of cigarette smoking on KIRC progression needed a further study.

Cigarette smoke contains numerous mutagens and carcinogens, such as nicotine, nitrosamines, polycyclic aromatic hydrocarbons, aromatic amines, and volatile organic compounds^9^. Nicotine is a major agent in cigarette smoke, but the carcinogenic effect of nicotine is intensified by converting to nicotine-derived nitrosamines^10^. 4-(methylnitrosamino)-1-(3-pyridyl)-1-butanone (NNK), the major ingredient and the most potent carcinogen among nicotine-derived nitrosamines, has reported to have relatively high levels in cigarette smoke and the smokers^11–14^. In this study, NNK was used to stimulate the RCC cells, and then the RNA-seq was performed in the KIRC cell line to identify the hub genes closely associated with NNK-induced malignancy. Next, the key candidate biomarkers were identified in the KIRC cohort of The Cancer Genome Atlas (TCGA) data. Thereafter, the gene signature was also constructed and its relationship some clinical traits and overall survival of KIRC patients was confirmed. Our work yielded a novel gene signature which was associated with tobacco smoke exposure and can accurately predict its relationship with KIRC progression.

## Results

### NNK exposure increased growth and migration abilities of RCC cells

**Figure 1:**
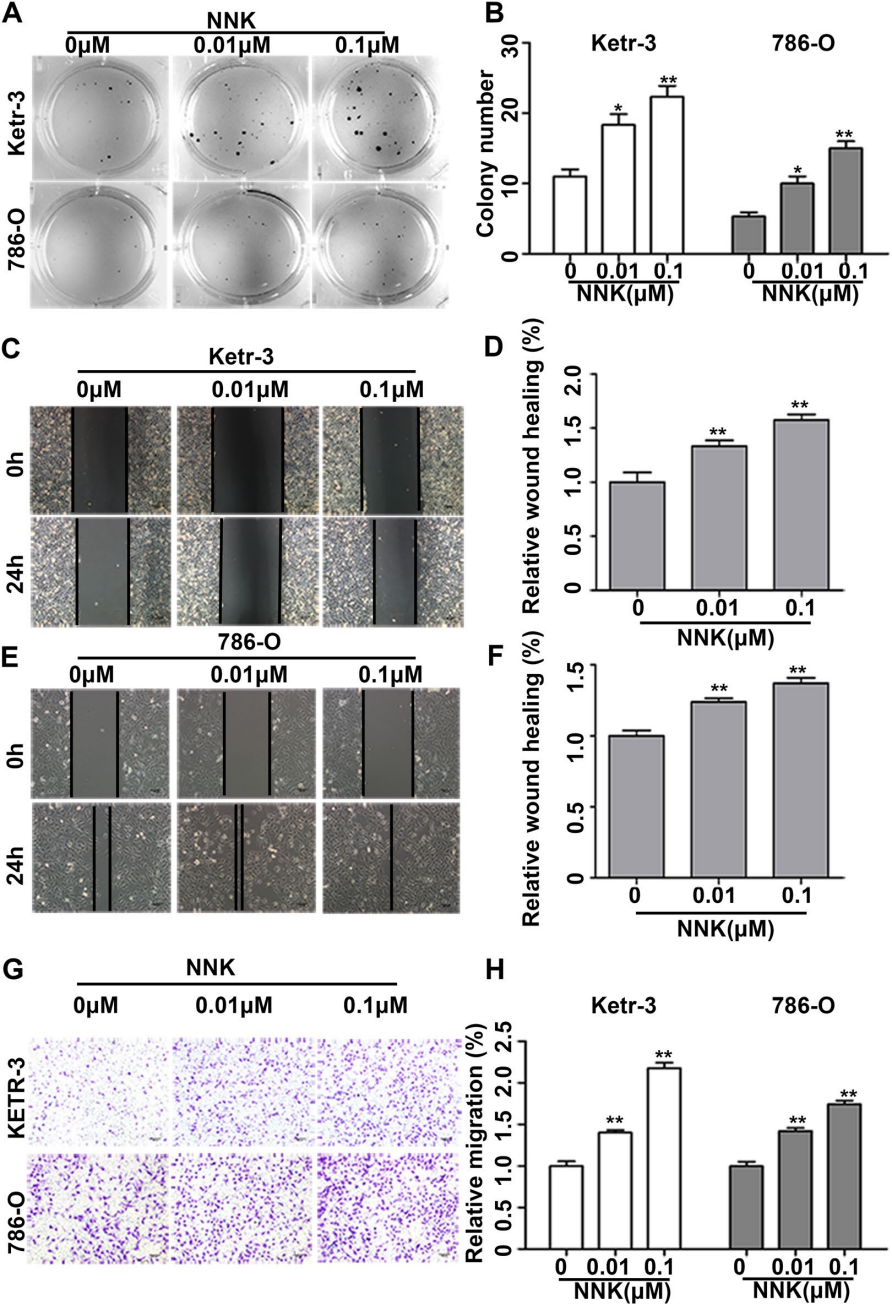
NNK Exposure Increased Growth and Migration Abilities of RCC Cells. (**A** and **B**) The soft agar colony formation of KETR-3 and 786-O cells exposed to 0 (0.1% DMSO), 0.01, 0.1 μM NNK at passage 40 for 120 days, and the number of cell colonies was counted (n = 3/group). (**C**–**F**) Cell wound healing assays in KETR-3 and 786-O cells exposed to 0 (0.1% DMSO), 0.01, 0.1 μM NNK at passage 40. The width of cell wound healing was measured (n = 3/group). (**G** and **H**) The migration of KETR-3 and 786-O cells exposed to 0 (0.1% DMSO), 0.01, 0.1 μM NNK at passage 40 (magnification × 100), and the relative number of cell migration per field was showed (n = 3/group). Data were presented as means ± standard deviations ^*^P < 0.05, ^**^P < 0.001.

The human RCC cell lines 786-O and KETR-3 were continuously exposed to 0.1% DMSO and NNK (0.01 and 0.1 μM) for 120 days (40 passages). Then the results of soft agar colony formation assay revealed that the number of cell colonies had a significant dose-dependent increase after NNK exposure when compared with the 0.1% DMSO control group (Fig. 1A and B). The wound healing assay was performed and showed that long-term NNK exposure significantly promoted KETR-3 and 786-O cell migration ability (Fig. 1C–F). The cell transwell assay also was performed and observed that the number of cell migration was significantly increased in a dose-dependent manner after long-term NNK exposure in both KETR-3 and 786-O cells when compared with the respective 0.1% DMSO controls (Fig. 1G and H).

### Identification of cigarette smoke exposure-related genes in RCC cells

**Figure 2:**
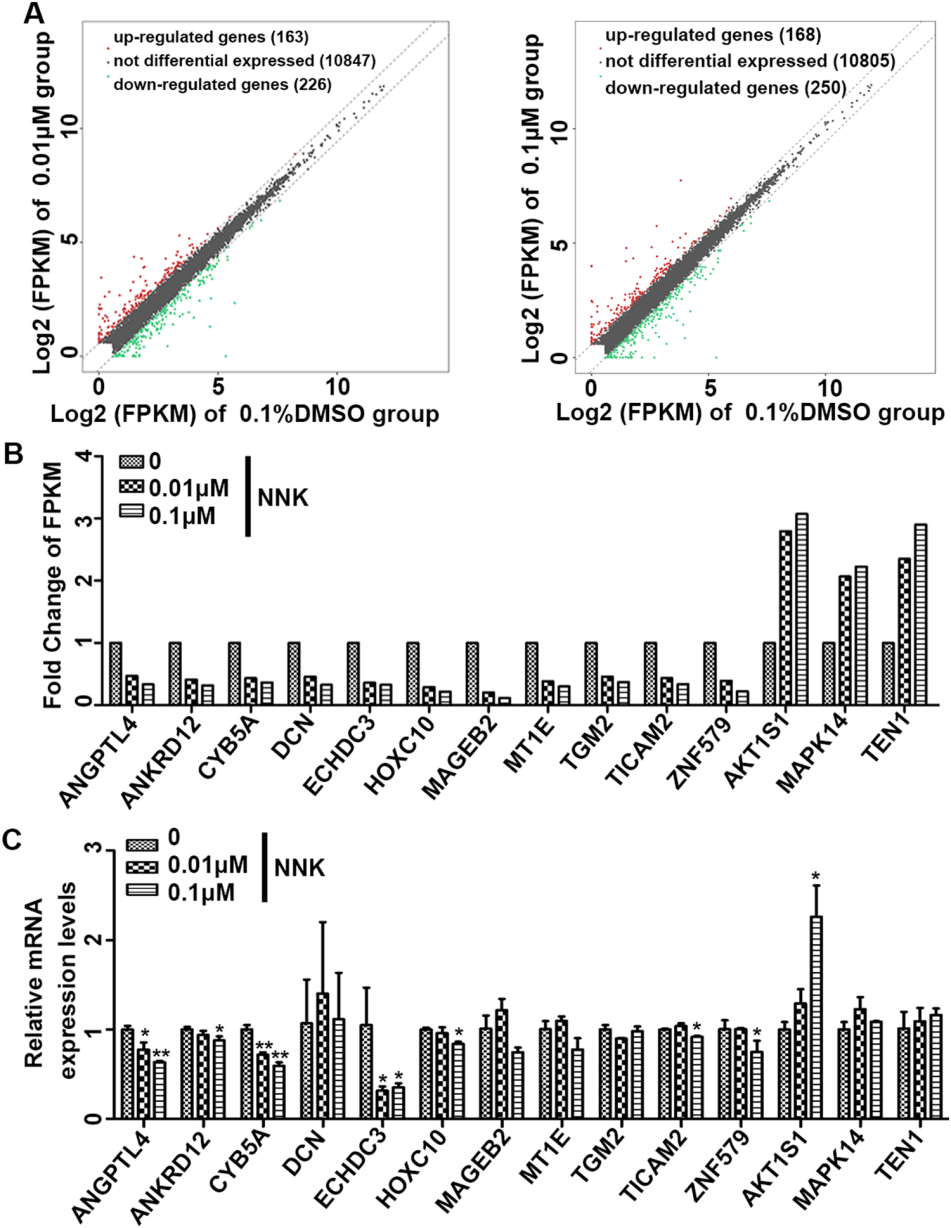
Identification of Cigarette Smoke Exposure-related Genes in RCC Cell. (**A**) The volcano plots of differentially expressed genes of 786-O cells exposed to 0 (0.1% DMSO), 0.01, 0.1 μM NNK at passage 40. (**B**) Identification of fourteen hub differentially expressed cigarette smoke exposure-related genes. (**C**) Real-time PCR validated the mRNA expression levels of fourteen hub differentially expressed cigarette smoke exposure-related genes in 786-O cells exposed to 0 (0.1% DMSO), 0.01, 0.1 μM NNK at passage 40. Data were presented as means ± standard deviations ^*^P < 0.05, ^**^P < 0.001.

We performed the RNA-seq in 0.1% DMSO, 0.01 μM, 0.1 μM NNK treated 786-O cells to explore the potential molecular mechanism in NNK-induced malignancy of RCC cells. The volcano plot identified 389 differentially expressed genes (163 up-regulated and 226 down-regulated genes) in 0.01 μM NNK-exposed group (Supplementary Table S1) and 418 differentially expressed genes (168 up-regulated and 250 down-regulated genes) in 0.1 μM NNK-exposed group (Supplementary Table S2) when compared with 0.1% DMSO group using the criteria of |log2(FC)|≥ 0.585 (Fig. 2A). Among all the differentially expressed genes, we found that eleven genes ANGPTL4, ANKRD12, CYB5A, DCN, ECHDC3, HOXC10, MAGEB2, MT1E, TGM2, TICAM2, ZNF579, and three genes AKT1S1, MAPK14, TEN1 were down-regulated or up-regulated in a NNK dose-dependent way with a cutoff criteria of |log2(FC)|≥ 1 and |log2(FC)_(0.1 μM NNK vs. 0.1% DMSO)_-log2(FC)_(0.01 μM NNK vs. 0.1% DMSO)_|≥ 0.1 (Fig. 2B). Real time PCR assay was used to validate the findings of RNA-seq in 786-O cells, our data showed that seven genes ANGPTL4, ANKRD12, CYB5A, ECHDC3, HOXC10, TICAM2 and ZNF579 were significantly down-regulated while AKT1S1 was significantly up-regulated after 0.1 μM NNK stimulation; in addition, two genes MAGEB2 and MT1E were marginally down-regulated in a after NNK exposure (Fig. 2C).

### Expression of fourteen cigarette smoke exposure-related genes in KIRC and normal kidney tissues in TCGA-KIRC dataset

**Figure 3:**
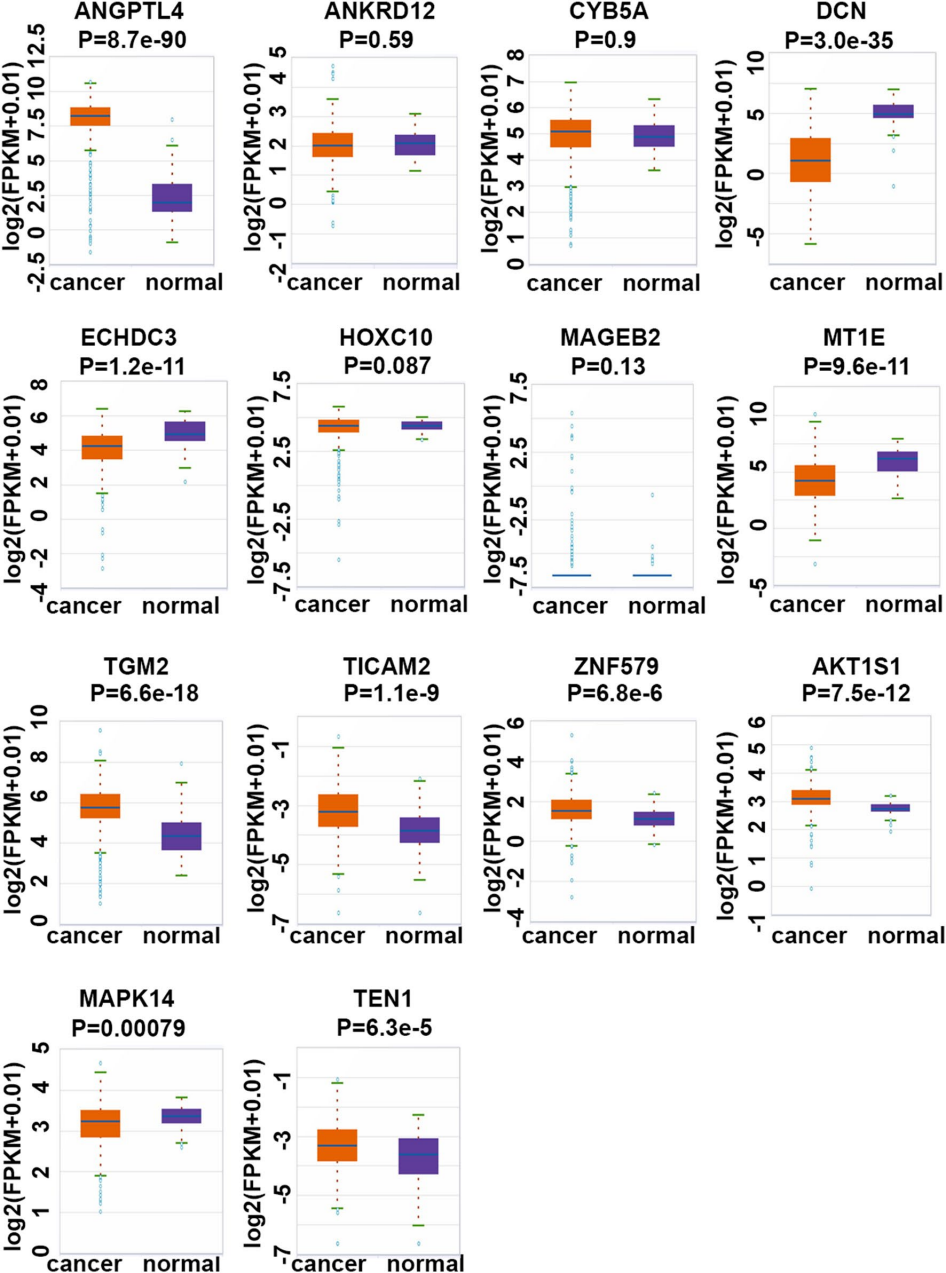
Expression of Fourteen Cigarette Smoke Exposure-related Genes in KIRC and normal kidney tissues in TCGA-KIRC dataset. The box plots of ANGPTL4, ANKRD12, CYB5A, DCN, ECHDC3, HOXC10, MAGEB2, MT1E, TGM2, TICAM2, ZNF579, AKT1S1, MAPK14, and TEN1 in KIRC tumor tissues (cancer, n = 531) and kidney normal tissues (normal, n = 72) from starBase database. Note: The gene-level transcription estimates was showed in a form of log2 (FPKM + 0.01).

To explore the roles of these fourteen genes in KIRC, we evaluated their expression patterns in TCGA-KIRC cohort. As shown in Fig. 3, we found that three cigarette smoke exposure-reduced genes DCN, ECHDC3, MT1E and two cigarette smoke exposure-induced genes AKT1S1, TEN1 were significantly down-regulated and up-regulated in KIRC when compared with the kidney normal tissues, respectively. Studies have reported that active smoking is associated with histological KIRC subtype^3,4^. Here we found that ECHDC3 expression in KIRC was continuously reduced, while AKT1S1 expression was gradually increased with the elevated malignancy of pathological grades (Supplementary Fig. S1). However, four cigarette smoke exposure-reduced genes ANGPTL4, TGM2, TICAM2, ZNF579, and one cigarette smoke exposure-promoted gene MAPK14 were significantly up-regulated and down-regulated in KIRC when compared with the kidney normal tissues, respectively (Fig. 3). Furthermore, TGM2 and TICAM2 expression in KIRC were gradually increased while MAPK14 expression was gradually decreased with the elevated malignancy of pathological grades (Supplementary Fig. S1). In addition, MAGEB2 had very low expression levels in KIRC and kidney normal tissues.

### Prognostic value of individual cigarette smoke exposure-related gene in TCGA-KIRC cohort

**Figure 4:**
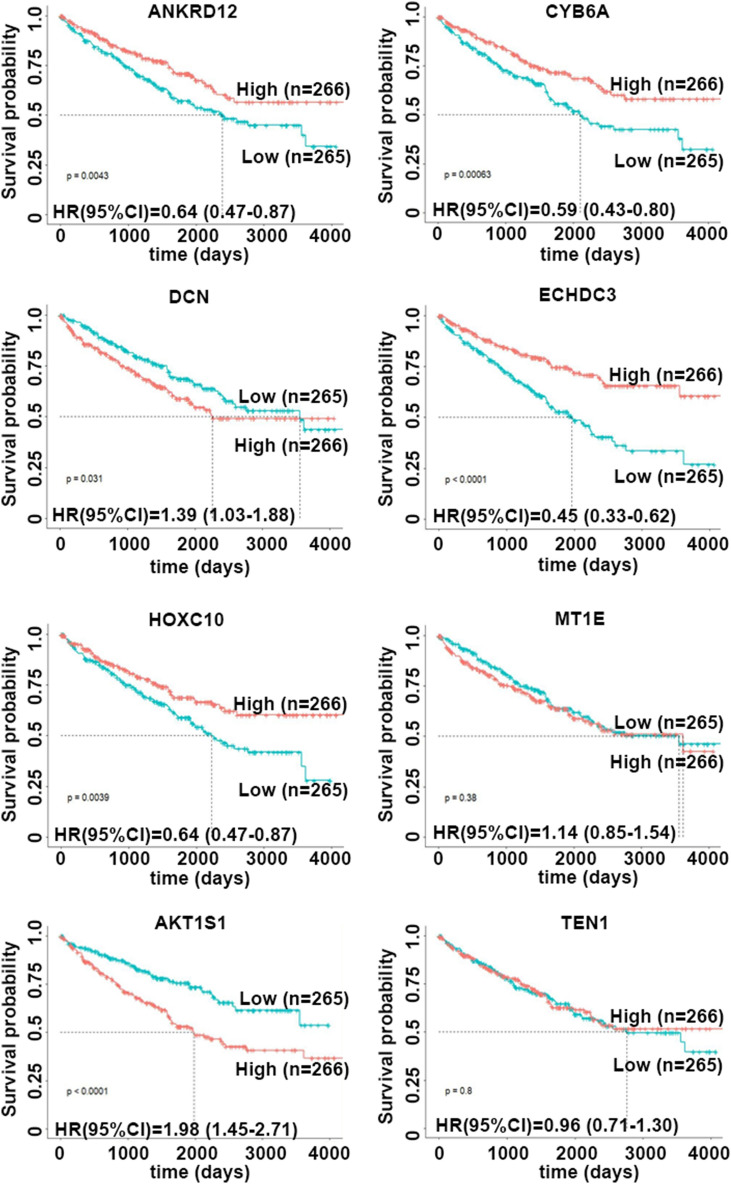
Prognostic Value of Individual Cigarette Smoke Exposure-related Gene In TCGA-KIRC Cohort. The Kaplan–Meier curves showed that the overall survival of patients with high or low expression (ANKRD12, CYB5A, DCN, ECHDC3, HOXC10, MT1E, AKT1S1, TEN1) based on defined cutoff (< or ≥ median of gene expression in cancer tissues) in TCGA-KIRC cohort.

Considering the opposite expression patterns in RNA-seq of NNK-stimulated cells and TCGA dataset and low gene expression of MAGEB2 in tissues, subsequently, eight cigarette smoke exposure-related genes ANKRD12, CYB5A, DCN, ECHDC3, HOXC10, MT1E, AKT1S1, TEN1 were selected to determine their prognostic value in TCGA-KIRC cohort. Each gene was classified as low or high expression based on defined cutoff (< or ≥ median of gene expression in cancer tissues). The Kaplan–Meier curves and univariate COX proportional regression models showed that ANKRD12 (HR (95%CI): 0.64 (0.47–0.87)), CYB5A (HR (95%CI): 0.59 (0.43–0.80)), ECHDC3 (HR (95%CI): 0.45 (0.33–0.62)), and HOXC10 (HR (95%CI): 0.64 (0.47–0.87)) had significantly positive while DCN (HR (95%CI): 1.39 (1.03–1.88)) and AKT1S1 (HR (95%CI): 1.98 (1.45–2.71)) had significantly negative relationship with overall survival of KIRC patients (Fig. 4).

### Construction and prognostic value of cigarette smoke exposure-related gene signature in TCGA-KIRC cohort

Individual KIRC tissue recently has been identified to have the substantial intratumour heterogeneity, demonstrating that single gene are unlikely to reveal a complete status of KIRC progression^15^. In addition, studies have found that the gene signature will be better than a single gene to judge prognosis of a variety of tumors^16–18^. Therefore, a single gene was not sufficiently comprehensive and efficient to evaluate the contribution of cigarette smoking to KIRC progression, in this study, the cigarette smoke exposure-related gene signature was produced through integrating multiple candidate genes. Lasso regression analysis was firstly used to avoid over-fitting problems in the gene signature, and five cigarette smoke exposure-related candidate genes ANKRD12, CYB5A, ECHDC3, MT1E, and AKT1S1 were retained when the optimal λ value was achieved (Supplementary Fig. S2A and B). Finally, a cigarette smoke exposure-related five-gene signature was established using the multivariate COX regression model and was digitized into a risk score based on the sum of the product of risk coefficient of each gene and the relevant mRNA expression level (Table 1).

**Table 1:** Genes included in prognostic gene signature.

Gene	Coefficient	*P* value
ANKRD12	− 0.12685	0.46205
CYB5A	− 0.29841	0.00180
ECHDC3	− 0.10969	0.20545
MT1E	0.04216	0.34586
AKT1S1	0.61537	0.00386

**Figure 5:**
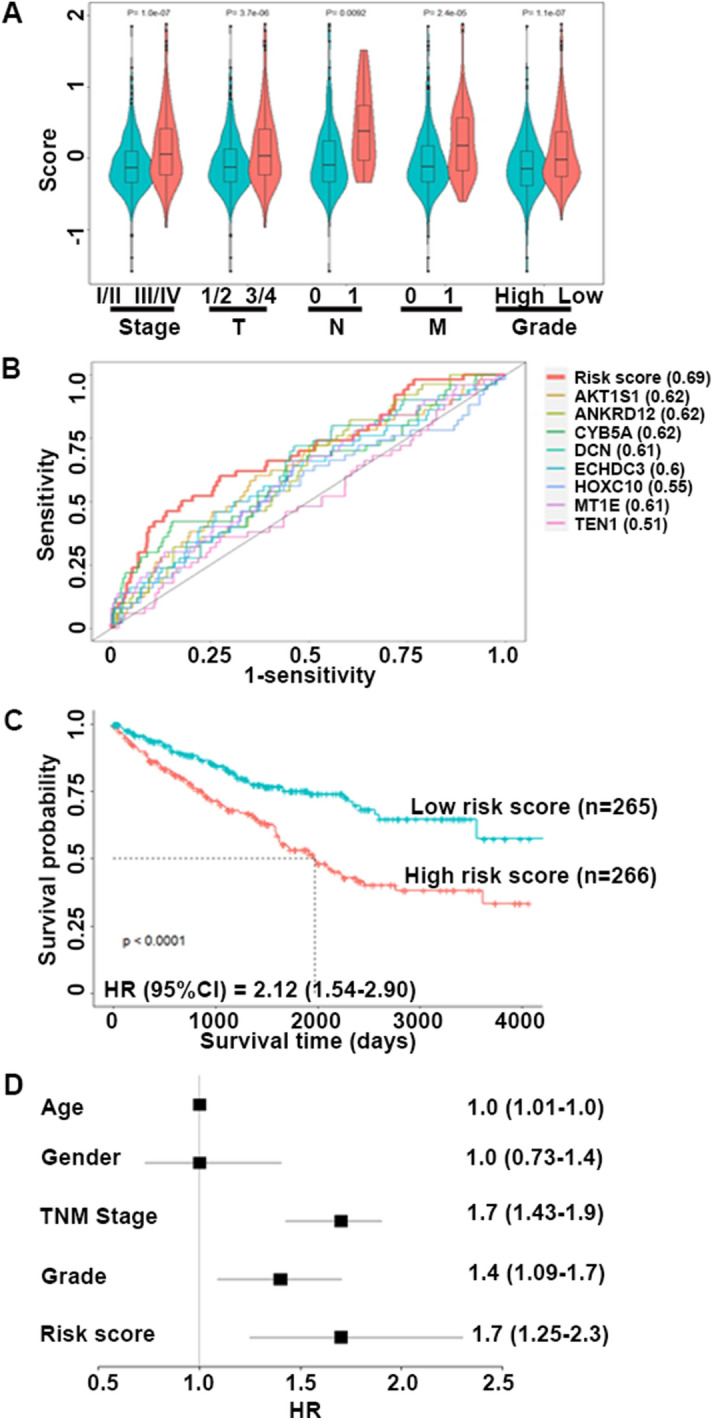
Prognostic Value of Cigarette Smoke Exposure-related Gene Signature in TCGA-KIRC Cohort. (**A**) Violin plots of risk score in the groups of TNM stage (I/II vs. III/IV) (n = 323 and 205 for stageI/II and III/IV); invasion depth (T1/2 vs. T3/4) (n = 341 and 190 for T1/2 and T3/4), lymph node metastasis (N0 vs. N1) (n = 239 and 16 for N0 and N1), and distant metastasis (M0 vs. M1) (n = 422 and 78 for M0 and M1), pathological grade groups (high vs. low) (n = 243 and 280 for high and low); (**B**) Time-dependent ROC curves of individual genes and gene signature for 1-year overall survival; (**C**) Kaplan–Meier curves showed that the overall survival of patients with high or low gene signature defined cutoff (< or ≥ median risk score) in TCGA-KIRC cohort; (**D**) Forest plot of hazard ratios of clinicopathological features and gene signature (risk score) for overall survival of KIRC in multivariate COX regression model. ^*^P < 0.05, ^**^P < 0.001.

We examined the correlation of the risk score with patients’ clinicopathlogical characteritics, and found that the risk score was significantly higher in advanced TNM stage (III/IV), invasion depth T3/4, lymphatic node metastasis N1, distant metastasis M1 and low pathological grade groups (G3/G4) when compared with early TNM (I/II), T1/2, N0, M0 and high pathological grade groups (G1/G2) (Fig. 5A). The time-dependent ROC curve was used to identify predictive value for KIRC patients’ survival and revealed that the risk score had a larger area under the curve (AUC) than individual genes (Fig. 5B).

Next, patients were classified to low-risk or high-risk group based on the median threshold of the risk score to further explore the prognostic value of the risk score in KIRC (Supplementary Fig. S2C). We found that the number of deaths was significantly much more in the high-risk group than the low-risk group, and survival time of the death sample significantly decreased with the decreasing risk score (Supplementary Fig. S2D). The Kaplan–Meier curve showed that the survival time of patients with high-risk score was significantly shorter than the time of patients with lower-risk score (HR = 2.12, 95%CI: 1.54–2.90) (Fig. 5C). In addition, patients with high-risk score was a significantly adverse prognostic indicator in both early and advanced TNM stages (Supplementary Fig. S3). Multivariate analysis showed that the risk score was an independent prognostic indicator (HR = 1.7, 95%CI: 1.25–2.30) after adjusting with age, sex, tumor TNM stage and grade (Fig. 5D).

### Nomogram construction and validation

**Figure 6:**
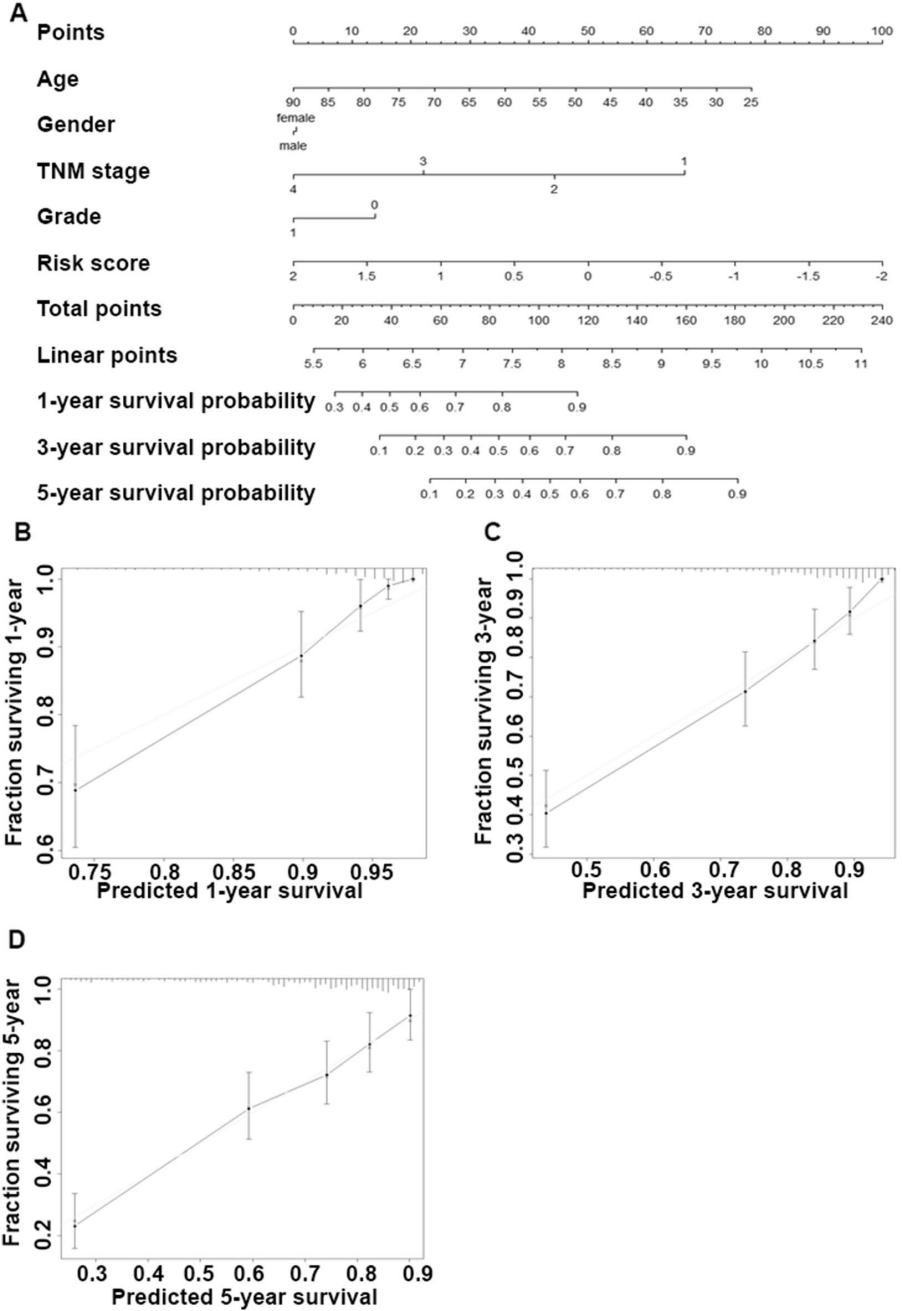
Nomogram Construction and Validation. (**A**) The nomogram for quantitatively predicting 1-, 3-, and 5-year overall survival of patients in TCGA-KIRC cohort (n = 531); (**B**–**D**) Calibration curve of the 1-, 3-, and 5-year overall survival.

Based on the multivariate COX proportional regression model (Fig. 5D), the prognostic nomogram was constructed to quantitatively predict the individualized prognostic risk for 1-, 3-, and 5-year overall survival by integrating cigarette smoke exposure-related gene signature risk scores with baseline variables (age and gender) and other independent clinical variables (grade and TNM stage). Each variable was assigned a corresponding point value based on its risk contribution to this model (Fig. 6A). Finally, the calibration curves suggested the agreement between the actual and predicted overall survival. The calibration curve showed that the 1-, 3-, and 5-year overall survival predicted by the nomograms were consistent with actual observations (Fig. 6B–D), indicating that the nomograms performed well.

### NNK exposure promoted AKT1S1 expression and activated AKT/mTOR signaling pathways

**Figure 7:**
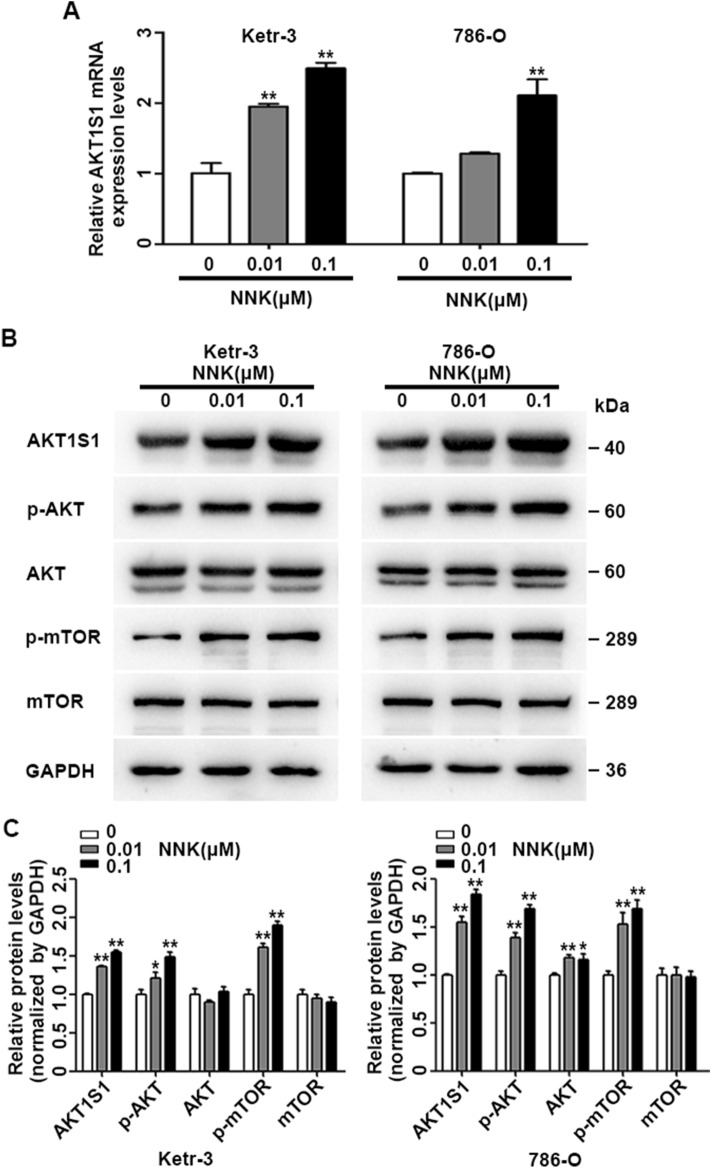
NNK Exposure promoted AKT1S1 expression and activated AKT/mTOR signaling pathway. (**A**) Real time PCR assay showed the relative mRNA expression levels of AKT1S1 in Ketr-3 and 786-O cells exposed to 0.01, 0.1 μM NNK when compared with 0 (0.1% DMSO) exposure at passage 40; (**B**–**C**) The protein and relative expression levels of AKT1S1, p-AKT, AKT, p-mTOR, mTOR, GAPDH in Ketr-3 and 786-O cells exposed to 0 (0.1% DMSO), 0.01, 0.1 μM NNK at passage 40. ^*^P < 0.05, ^**^P < 0.001.

In this study, we found that AKT1S1 played as the most important component in the cigarette smoke exposure-related gene signature; and studies have showed that AKT1S1 acts an critical role in the intersection of the AKT/mTOR signaling pathways^19^, therefore, we explored AKT1S1 expression level in Ketr-3 and 786-O cells exposed to 0.1% DMSO and 0.01, 0.1 μM NNK. We found that NNK exposure dramatically up-regulated mRNA levels of AKT1S1 (Fig. 7A), and AKT1S1, p-AKT, p-mTOR protein levels had significant increase after NNK exposure when compared with the 0.1% DMSO control group (Fig. 7B–C). In addition, in order to detect the function and pathways of AKT1S1 in NNK-promoted KIRC progression, we constructed the protein interaction network (PPI network) with the String database (https://string-db.org/) (Supplementary Fig. S4). The results of showed that AKT1S1 was enriched in mTOR signaling pathway with the lowest false discovery rate value in the biological process of Gene Ontology (GO) analysis, and in autophagy pathway with the lowest false discovery rate value in the KEGG analysis.

## Discussion

It’s well-known that tumor cells infinite growth and metastasis are the crucial characters of tumor malignant progression^20^. Epidemiological data have indicated that cigarette smoking is associated with the tumor malignant progression and the poor prognosis of KIRC^21,22^. In this study, we found that long-term exposure to the major component of cigarette smoke, nicotine-derived NNK, increased the abilities of RCC cells colony formation and migration, which were key events of tumor growth and metastasis. Based on these findings, it was not surprising to see that cigarette smoke enhanced the malignant phenotypes of tumor cells to eventually promote KIRC progression.

There are multiple molecular events that cigarette smoke initiates and promotes the malignancy of RCC. Recently the relevance of cigarette smoke carcinogens with the inactivation of tumor suppressor genes or the activation of oncogenes has been validated for the development and progression of cancer^23,24^. Thus, further exploration of the molecular events involved in NNK-induced malignancy of RCC cells might provide new biomarkers for progression of KIRC. Here, we performed the genome-wide sequencing to seek potential biomarkers and found fourteen cigarette smoke exposure-related genes showing NNK dose-dependent down-regulation or up-regulation. Moreover, the real time PCR validated the reliability of RNA-seq.

Therefore, we reasonably speculated that cigarette smoke exposure-related genes have broad prospects in progression evaluation of KIRC. Through compared the gene expression patterns in KIRC and normal kidney tissues, we found nine genes were not inconsistent with the findings in RCC cells with NNK exposure. Next, the survival analysis indicated that cigarette smoke exposure-reduced genes ANKRD12, CYB5A, ECHDC3, DCN, HOXC10 and cigarette smoke exposure-promoted gene AKT1S1 showed the positive and negative relationship with overall survival in KIRC, which was accord with some studies that decreased ANKRD12 and CYB5A and increased AKT1S1 expression show a higher frequency of tumor metastasis and are indicators of increased risk of tumor progression^25–27^. Though cigarette smoke exposure-reduced DCN was found to be negative prognostic factor in KIRC, increasing evidences indicate that lack of DCN expression has been regarded as an indicator of tumor metastasis^28^.

However, cancer heterogeneity leads to unsatisfactory effects of individual genes on the progression judgment in KIRC patients. Therefore, new efforts are urgently required to develop comprehensive estimate for KIRC. Studies have found that the gene signature will be better than a single gene to judge prognosis of a variety of tumors^16–18^. In this study, Lasso regression was used to screen variables to establish the prognostic model to avoid extreme prediction. The new cigarette smoke-related five-gene signature was established using the multivariate COX regression model. To provide a clinically quantitative method for gene signature, we produced a risk score based on risk coefficient of each gene and the relevant mRNA expression level. This scoring approach and its cut-off value have been confirmed to be robust in some cancer-related studies, which may be readily translated to clinical practice^29–31^.

Our data showed that the gene signature with high-risk score was significantly associated with the increased tumor invasion depth, lymphatic node metastasis, and distant metastasis and advanced TNM stage. Using the time-dependent ROC curve, we found that the risk score had a better predictive value than individual genes in KIRC prognosis. More importantly, the risk score significantly stratified patient outcomes and high-risk score was a significantly more unfavorable factor for KIRC prognosis than any single gene, indicating that the risk score had a stronger prognostic power than single genes.

Considering that AKT1S1 was the most important component of the cigarette smoke exposure-related gene signature, here significantly up-regulated AKT1S1 expression was observed after NNK exposure, which was accord with some reports that elevated AKT1S1 expression in cancer cells and could contribute to tumor metastasis^27,32^. Studies have showed that AKT1S1 is involve in regulating cell growth, cell apoptosis, oxidative stress, autophagy and angiogenesis through various of signaling pathways such as AKT, mTOR, NF-κB and et al.; AKT1S1 phosphorylation state could predict hyperactivation of the AKT/mTOR pathway in multiple cancer cell types^19,33^. In this study, we found that NNK exposure activated AKT/mTOR signaling pathway. And the protein interaction network (PPI network) showed that the function and pathways of AKT1S1 were mainly enriched in mTOR and autophagy pathway. In addition, studies have identified AKT/mTOR signaling as important dysregulated pathways in KIRC, and some mTOR targeted inhibitors, such as everolimus and temsirolimus, have been validated to contribute to better clinical outcome of metastatic renal cell carcinoma^34–36^. Therefore, our data suggested that the up-regulation of AKT1S1, AKT/mTOR signaling pathway and autophagy might play an important role in cigarette smoke-induced KIRC metastasis and progression (Supplementary Fig. S5).

In summary, NNK exposure promoted the growth and migration abilities of RCC cells. Using RNA-seq, fourteen cigarette smoke exposure-related genes were obtained. The expression patterns showed that nine genes in KIRC when compared with normal kidney tissues were not inconsistent with the findings in RCC cells with NNK exposure, and their prognostic value were further analyzed. Five cigarette smoke-related gene signature was screened and integrated by Lasso regression analysis and multivariate COX regression model. The gene signature was more powerful than any signal gene for predicting the prognosis of KIRC patients. Moreover, NNK exposure-induced AKT1S1 and its related AKT/mTOR signaling pathway might play an important role in cigarette smoking-induced KIRC progression. Therefore, our findings provided a significant mechanistic insight into cigarette smoke-induced KIRC progression and supported that the cigarette smoke-related gene signature might serve as a highly efficient biomarker to identify metastasis and prognosis of KIRC patients.

However, a limitation is the lack of animal models of NNK-promoted KIRC progression and the validation of gene signature expression in NNK-promoted tumor tissues of animal models. In addition, this study is underpowered to assess the role of AKT1S1 and its related AKT/mTOR signaling pathway in NNK exposure-induced KIRC progression unless AKT1S1 inhibitors were used in NNK-stimulated cell and animal models.

## Methods

### Cell lines and reagent

Human KIRC cell line 786-O and another RCC cell line KETR-3 were purchased from the Shanghai Institute of Biochemistry and Cell Biology, Chinese Academy of Sciences (Shanghai, China). 786-O and KETR-3 cells were separately cultured in RPMI-1640 and DMEM medium supplemented with 10% fetal bovine serum (FBS), 100U/ml penicillin and 100 μg/ml streptomycin. Cells were grown at 37 °C in the presence of 5% CO_2_ in a humidified incubator. NNK was purchased from Sigma-Aldrich (CAS: 64091-91-4, St Louis, MO,).

### Soft agar colony formation assay

The 6-well plates were firstly coated with 0.60% agarose. Then 500 cells per well were plated in triplicate in 1 ml of 0.35% agarose over 0.60% agarose. Cultures were fed every 3 days. At 14 days, the 0.5% NBT was used to dye the colonies. Colonies which were dyed strongly brown were scored as “positive” and colony-forming number in each well was counted by Image J software.

### Wound healing assay

Cells were grown to 80% confluence into a 6-well plate in complete medium overnight and converted to serum-free medium for another 12 h at 37 °C and 5% CO_2_. An injury line was made using a 2-mm-wide plastic pipette tip. Then the wells were rinsed with phosphate-buffered saline and covered with serum-free medium, and the photographs were acquired at 0 h and 24 h, respectively. Then the scratch width of every group at 0 h and 24 h were measured, and migration distance was calculated through subtracting the scratch width at 24 h from the scratch width of 0 h. Finally the relative ratio of migrating distance in NNK groups and DMSO group was calculated.

### Transwell assay

The transwell filter inserts with a pore size of 8 μm were used for the cell migration assay. 2 × 10^4^ cells (for 786-O) or 5 × 10^4^ cells (for KETR-3) in serum-free medium were added in the upper chamber, and placed in 24-well plate containing 500 μl complete medium. After 12 h incubation at 37 °C, cells in the upper chamber were carefully removed with a cotton swab and the cells that had traversed the membrane were fixed in methanol, stained with crystal violet (0.04% in water; 100 μl). Then these inserts were placed under the inverted microscope (100 ×), and five fields of each insert were photographed. The crystal violet positive permeating cells of each field were counted by Image J software, and the relative ratio of migrating cells number in NNK groups and DMSO group was calculated.

### Transcriptome resequencing and quantitative analysis

Human transcriptome resequencing (Vazyme, China) was used to analyze gene expressions collected from 786-O cells which were exposed 0.1%DMSO, 0.01 μM, 0.1 μM NNK at 40 passages for 120 days. The Cufflinks (cufflinks-2.2.1) was used to perform the quantitative analysis of gene expression.

### Western blot analysis

Western blot was carried out as previously reported^37^. Total cell lysates were prepared with a detergent lysis buffer (#P0013B, Beyotime Biotechnology, China) and the protein concentration was measured with BCA Protein Assay Kit (#P0010, Beyotime Biotechnology, China). Equal amounts of protein from cell lysates were separated by SDS-PAGE, and transferred by electroblotting to a polyvinylidene fluoride membrane. After blocked with 5% nonfat milk in TBST buffer for 2 h, the membranes were cut according to the molecular weight of the antibody specification and the PageRuler™ Prestained Protein Ladder (#26616, Thermo Scientific, USA) on the membrane, then the membranes were incubated with primary antibodies, including anti-AKT1S1 (1:1000; #ab151719, Abcam, USA), anti-AKT (1:1000; #9272, CST, USA), anti-pAKT (1:1000; #4060, CST, USA), anti-mTOR (1:1000; #2972, CST, USA), anti-p-mTOR (1:1000; #5536, CST, USA) and anti-GAPDH (1:1000; #AF1186, Beyotime Biotechnology, China) overnight at 4 °C. Then, the membrane was incubated with anti-rabbit immunoglobulin G conjugated to horseradish peroxidase (1:2000; #A0208, Beyotime Biotechnology, China) for 2 h. The anti-GAPDH was used for the protein loading control. The antigen–antibody complex was detected by an enhanced chemiluminescence system. All the blots were cut prior to hybridization with antibodies during blotting. Moreover, we checked these same molecules in 786-O, Ketr-3 and ACHN cells, and the results showed that the same molecule was displayed at the same location on the membrane and all the molecular weights were consistent with the antibody specifications. In addition, there were few or almost no non-specific bands in all blots. In the supplementary Fig. S6, we provided the images of all blots as they are, with membrane edges visible, all the experiments were repeated three times.

### Real-time PCR analysis

Total cell RNA was extracted using TRIzol reagent (#R401-01, Vazyme, China) and the purity was checked by OD260/280 of RNA samples (> 1.8). Real-time PCR was carried out in triplicate with HiScript II one step qRT-PCR SYBR Green Kit (#Q221-01, Vazyme, China) according to the manufactory instruction. GAPDH mRNA was used as an internal control for each sample, and the Ct value for each sample was normalized to GAPDH mRNA. The complementary DNA was amplified with the following primers:

**Table:** 

DCN Forward	TCCGCTGTCAATGCCATCTTCG
DCN Reverse	GCAGGTCTAGCAGAGTTGTGTCAG
HOXC10 Forward	CCGCCTATCGCCTGGAACAAC
HOXC10 Reverse	GCAGCAGACATTCTCCTCCTTGAC
AKT1S1 Forward	GCCGTTGCCTCCACGACATC
AKT1S1 Reverse	TCATCCTCGTCCTCCTCGTTGTC
MAPK14 Forward	GGCTCCTGAGATCATGCTGAACTG
MAPK14 Reverse	AGTCAACAGCTCGGCCATTATGC
ZNF579 Forward	AAGGCCGAGCAGGAGGAAGAAG
ZNF579 Reverse	AGGCTGAGTGGTCTTGGCTGTC
TICAM2 Forward	TCCTGCCCTCTTTCTCTCTCTTGG
TICAM2 Reverse	CCCCTCTGTTGTATTGCTGTGCTC
TGM2 Forward	CACCAACAACACCGCTGAGGAG
TGM2 Reverse	CAGGTTGAGGTTGAGCAGGTACTTG
MAGEB2 Forward	AACGGCCACACTTACACCTTCATC
MAGEB2 Reverse	ATCACACCCAGGAGAGGCATCAG
ANGPTL4 Forward	AGACACAACTCAAGGCTCAGAACAG
ANGPTL4 Reverse	TCTAGGTGCTTGTGGTCCAGGAG
CYB5A Forward	TCAGAAGCACAACCACAGCAAGAG
CYB5A Reverse	AGTTCTCAGTAGCGTCACCTCCAG
TEN1 Forward	TCCAGCATCAGCAGGACAGAGG
TEN1 Reverse	TGTTCCAACAAGGGCAGGTTCATC
MT1E Forward	TCAGGTTGGGAGGGAACTCAAGG
MT1E Reverse	GAGAGGGAATGACACGGGCAATG
ECHDC3 Forward	GCTAGGAAGATCGCATCGCTGAG
ECHDC3 Reverse	CCTGGGAGGTGAGGTAGTAAGCC
ANKRD12 Forward	CCAGGAAACTCTTGTGCTCAGGATC
ANKRD12 Reverse	TCTGAAAGTGATTGGCTGGGGAAAG
GADPH Forward	GCCGGT- GCTGAGTATGTC
GAPDH Reverse	CTTCTGGGTGGCAGTGAT

### Statistical analysis

All the statistical analyses were performed by R (version 4.0.3) statistical software. The starBase project (http://starbase.sysu.edu.cn/panCancer.php) was used to analyze the gene expression pattern in KIRC and kidney normal tissues. The univariate and multivariate COX proportional regression models were performed to estimate the crude hazard ratios (HRs), adjusted HRs and their 95% confidence intervals (CIs). Lasso regression analysis was used to screen the prognostic genes. Operating characteristic curve (ROC) was used to predict the prognostic value of genes. The Kaplan–Meier method and log-rank test were used to test the differences in survival as a function of time between the low and high risk score groups. The nomogram was developed to predict survival probability, and the fitting degree of the nomogram was evaluated by calibrations. The ANOVA analysis or Student t-test was used to evaluate the significance of quantitative data. *P* value < 0.05 was deemed statistically significant, and all tests were two sided.

## Supplementary Information

Supplementary Information 1.

Supplementary Information 2.

Supplementary Information 3.

## Data Availability

The clinical features of KIRC patients can be obtained from TCGA database (https://portal.gdc.cancer.gov/, repository, cases: project (TCGA-KIRC)) and the corresponding RNA-sep data can be obtained downloaded from the UCSC Xena (https://gdc-hub.s3.us-east-1.amazonaws.com/download/TCGA-KIRC.htseq_fpkm.tsv.gz). Z.L. and Y.C. had full access to all of the data in the study and take responsibility for the integrity of the data and the accuracy of the data analysis. The datasets generated and/or analysed during the current study are available from the corresponding author on reasonable request.
