# Supplementary material for: Identification and validation of a cigarette smoke-related five-gene signature as a prognostic biomarker in kidney renal clear cell carcinoma
Source: Sci Rep. 2022 Feb 9;12:2189. doi: 10.1038/s41598-022-06352-y (PMC8828851; doi:10.1038/s41598-022-06352-y)
Supplement: Supplementary file 1 — Supplementary Information 1. [file 41598_2022_6352_MOESM1_ESM.pdf]

Identification and validation of a cigarette smoke-related five-gene signature as a prognostic biomarker in kidney renal clear cell carcinoma

Yefei Huang[1#], Qinzhi Wang[1#], Yu Tang[1], Zixuan Liu[1], Guixiang Sun[1], Zhaojun Lu[1,2*] and Yansu Chen[1*]

[1]Xuzhou Key Laboratory of Environment and Health, School of Public Health, Xuzhou Medical University, Xuzhou 221004, Jiangsu Province, China;

[2]Second Clinical Medical College, Xuzhou Medical University, Xuzhou 221004, Jiangsu Province, China

[#]Yefei Huang and Qinzhi Wang have contributed equally in this study.

[*]Corresponding authors

Correspondence and requests for materials should be addressed to Y.C. (100002014013@xzhmu.edu.cn)

Supplementary Figure Legends

Supplementary Figure S1. The box plots of ANGPTL4, ANKRD12, CYB5A, DCN, ECHDC3, HOXC10, MAGEB2, MT1E, TGM2, TICAM2, ZNF579, AKT1S1, MAPK14, and TEN1 in kidney normal tissues and KRIC tumor tissues with different pathological grades (n=72 for kidney normal tissues and n=13, 230, 206, and 74 for KRIC tumor tissues with different pathological grades G1, G2, G3 and G4). Note: The gene-level transcription estimates was showed in a form of log2 (norm_count +1) (hub: https://tcga.xenahubs.net).

Supplementary Figure S2. Construction and Risk Assessment of Cigarette Smoke Exposure-related Gene Signature in TCGA-KIRC Cohort. (A) Plots for Lasso expression coefficients of eight cigarette smoke exposure-related genes; (B) Cross-validation plot for the penalty term; (C) Distribution of low-risk or high-risk group based on the median risk score; (D) Survival status together with overall survival of low-risk and high-risk group.

Supplementary Figure S3. Prognostic Value of Cigarette Smoke Exposure-related Gene Signature in patients with early or advanced stage in TCGA-KIRC Cohort. (A) Kaplan-Meier curves of high or low gene signature defined cutoff (< or ≥ median risk score) in patients with early stage (Ⅰ/Ⅱ) in TCGA-KIRC cohort; (B) Kaplan-Meier curves of high or low gene signature defined cutoff (< or ≥ median risk score) in patients with advanced stage (Ⅲ/Ⅳ) in TCGA-KIRC cohort.

Supplementary Figure S4. Protein interaction network diagram of AKT1S1. The protein interaction network (PPI network) of AKT1S1 was constructed with the String database.

Supplementary Figure S5. The hypothesis mechanism figure of cigarette smoke-induced KIRC metastasis and progression.

Supplementary Figure S6. Images of all replicate blots of figure 7B. The blots were cut prior to hybridization with antibodies during blotting and the black box represents the edges of the original blots. The molecular size of the protein has been marked on the left of the black box according to the PageRuler™ Prestained Protein Ladder. (A) The first replicate blots of AKT1S1, p-AKT, AKT, p-mTOR, mTOR, GAPDH in KETR-3 and 786-O cells exposed to 0 (0.1% DMSO), 0.01, 0.1μM NNK at passage 40. (B) The second replicate blots of AKT1S1, p-AKT, AKT, p-mTOR, mTOR, GAPDH in KETR-3 and 786-O cells exposed to 0 (0.1% DMSO), 0.01, 0.1μM NNK at passage 40. (C) The third replicate blots of AKT1S1, p-AKT, AKT, p-mTOR, mTOR, GAPDH in KETR-3, 786-O and ACHN cells exposed to 0 (0.1% DMSO), 0.01, 0.1, 1μM NNK at passage 40.

Supplementary Table S1. List of differentially expressed genes in 786-O cells exposed to 0.01μM NNK group as compared to 0.1%DMSO group (shown in excel sheet).

Supplementary Table S2. List of differentially expressed genes in 786-O cells exposed to 0.1μM NNK group as compared to 0.1%DMSO group (shown in excel sheet).

Supplementary Figure S1

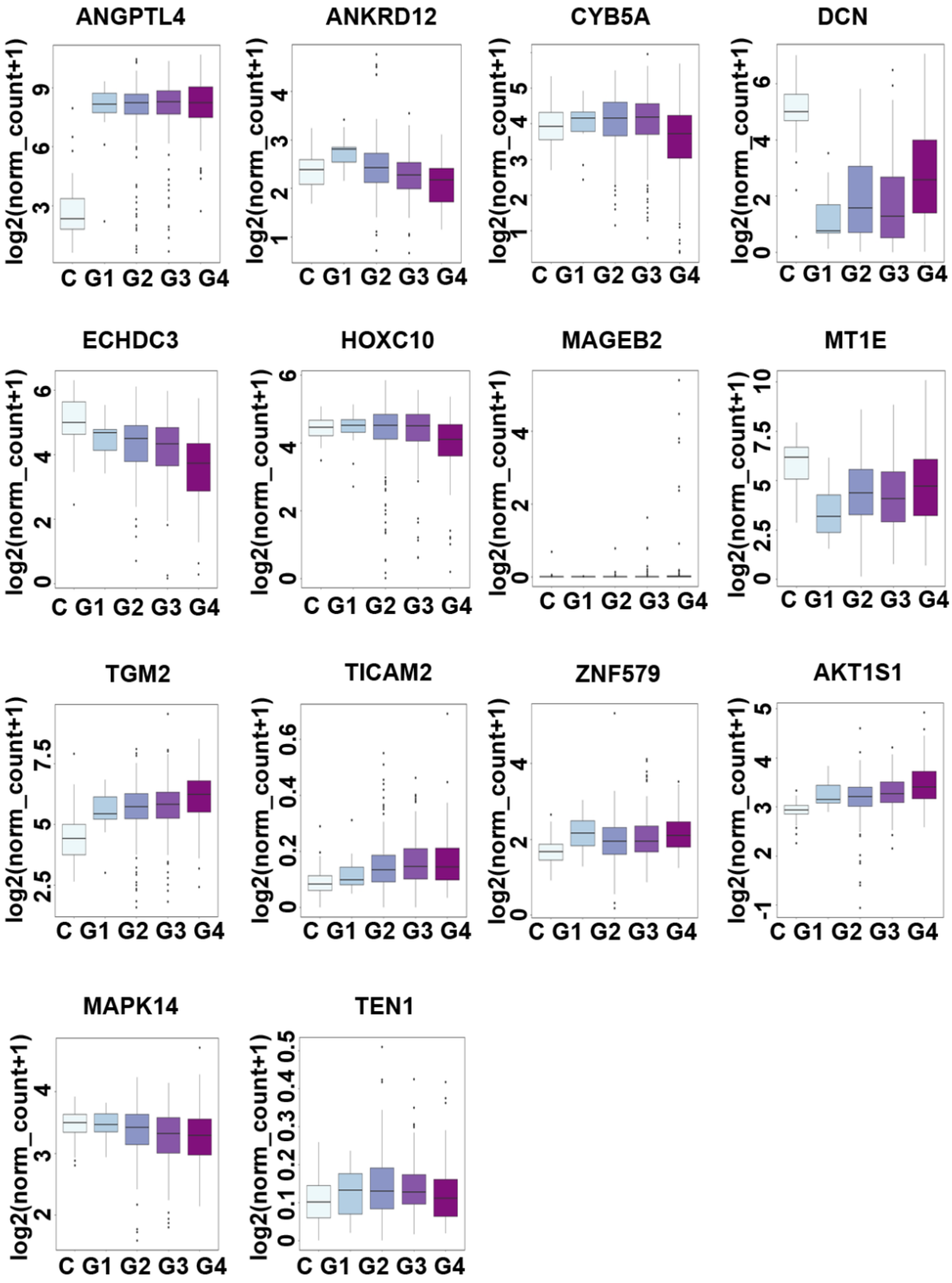

Supplementary Figure S2

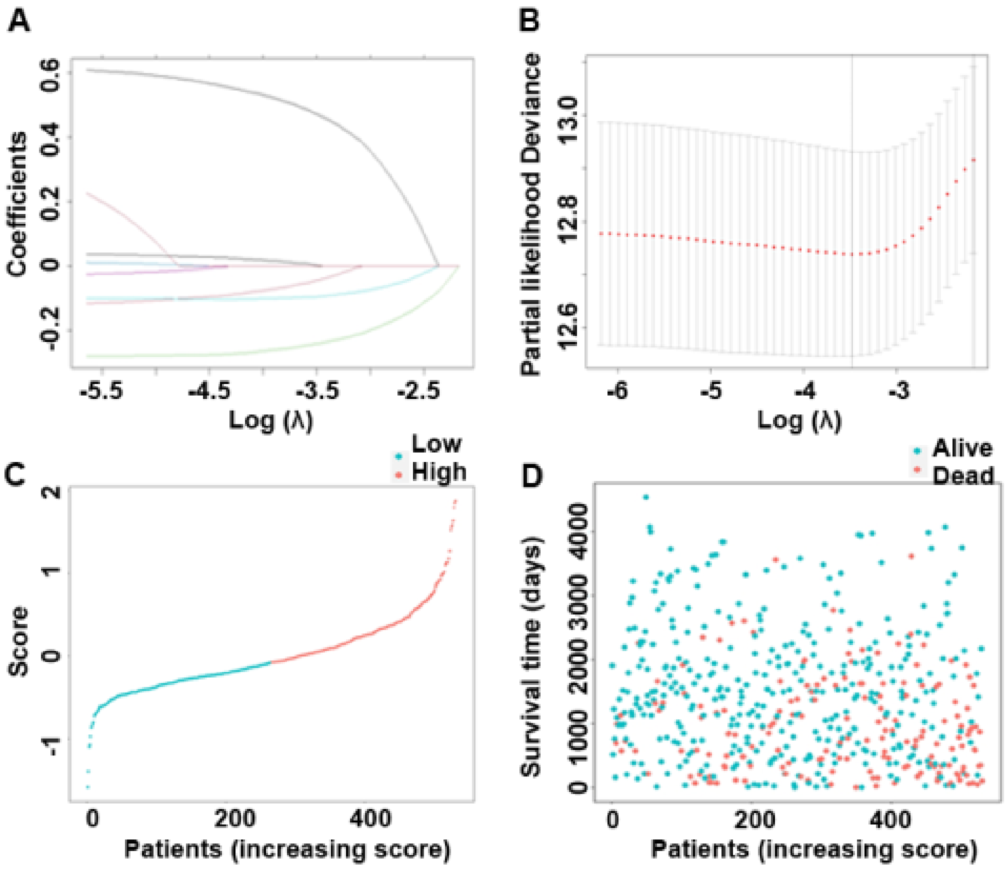

Supplementary Figure S3

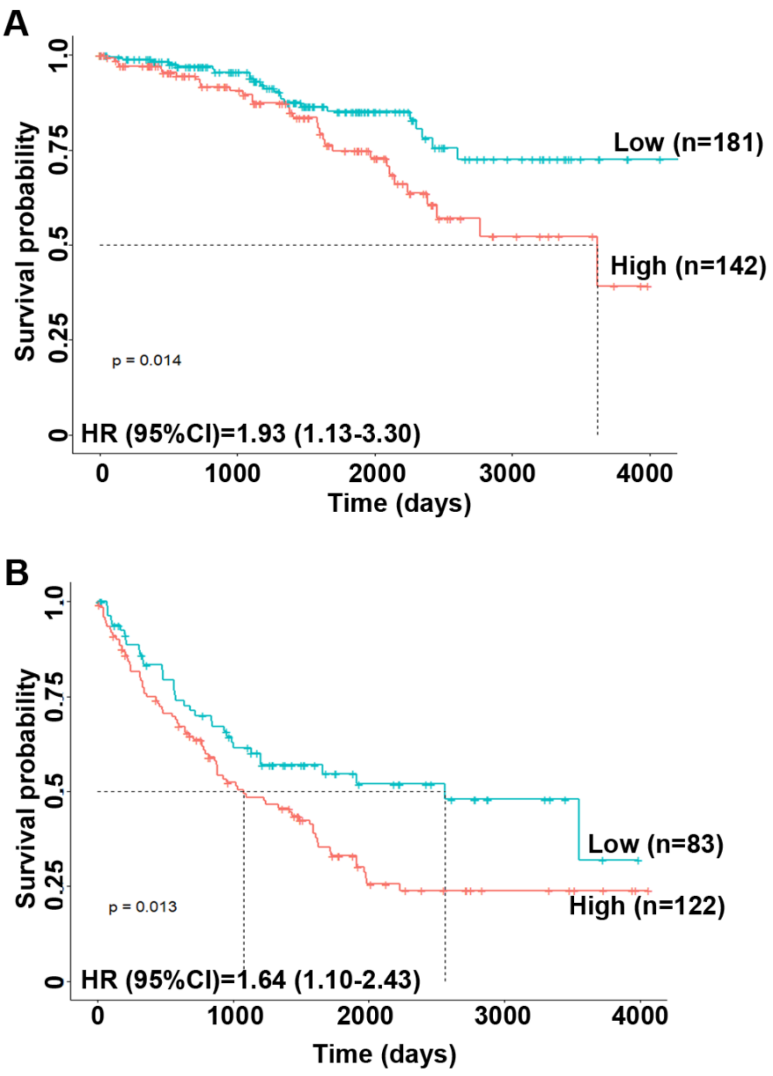

Supplementary Figure S4

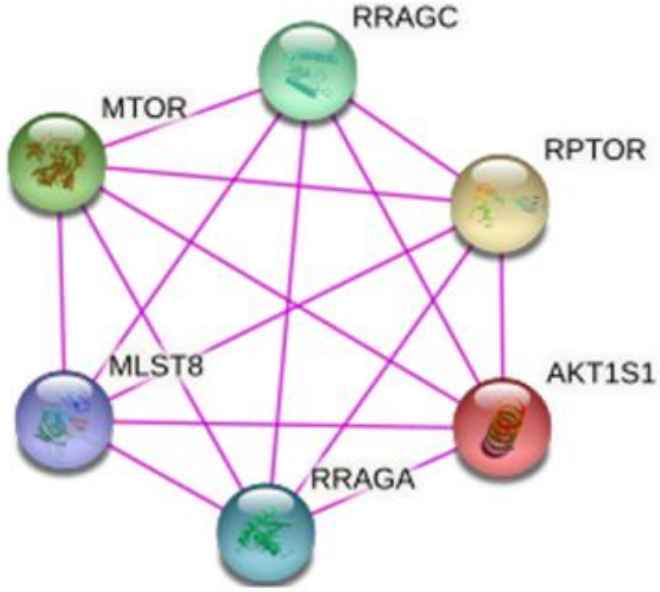

Supplementary Figure S5

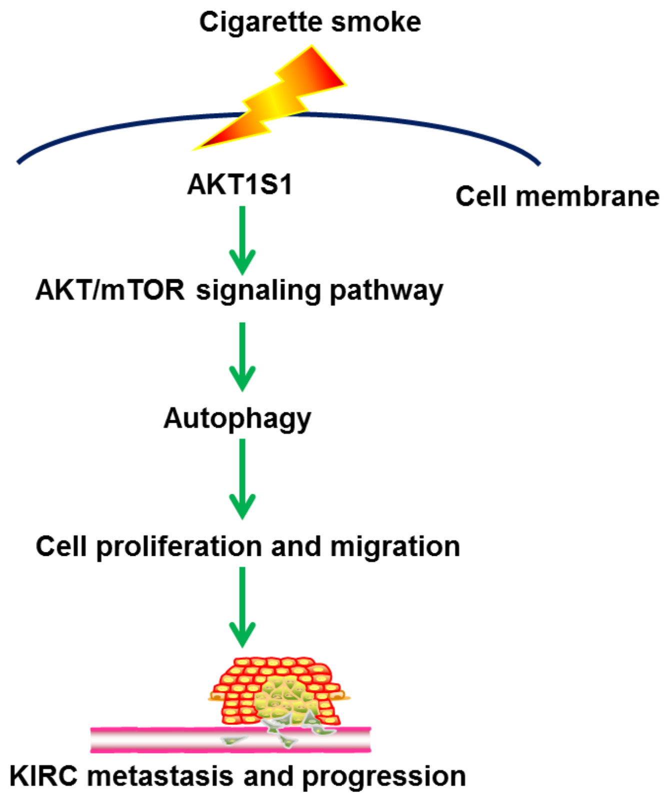

Supplementary Figure S6

A

The first replicate blots of figure 7B

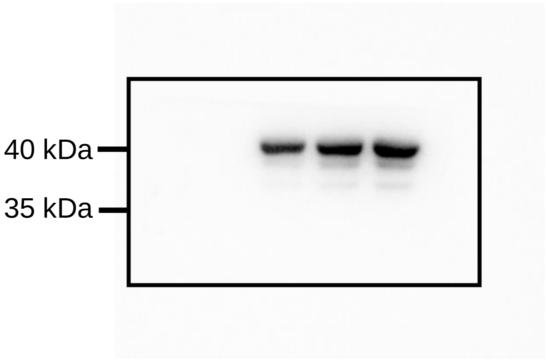

Ketr-3 AKT1S1

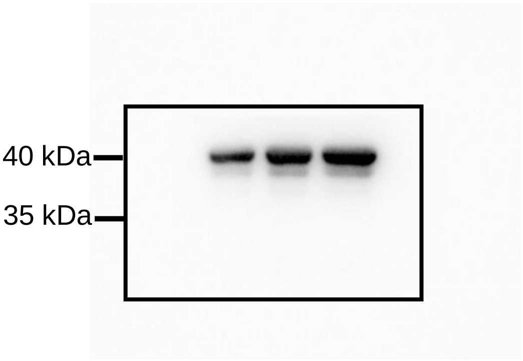

786-O AKT1S1

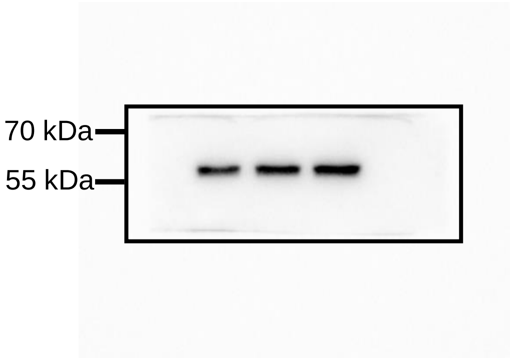

Ketr-3 p-AKT

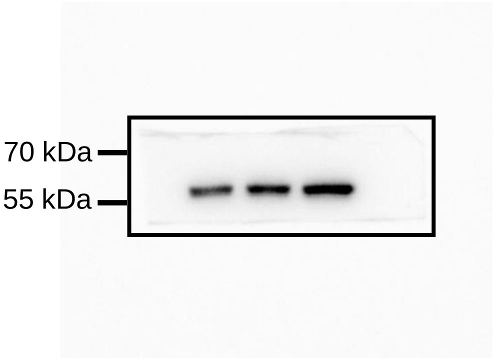

786-O p-AKT

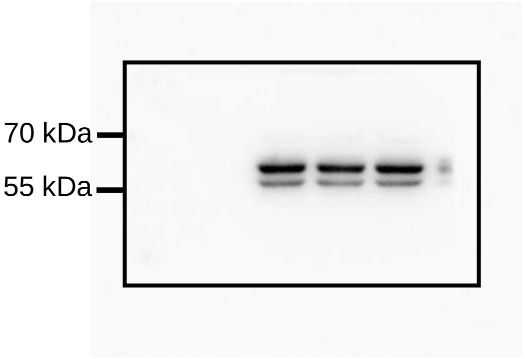

Ketr-3 AKT

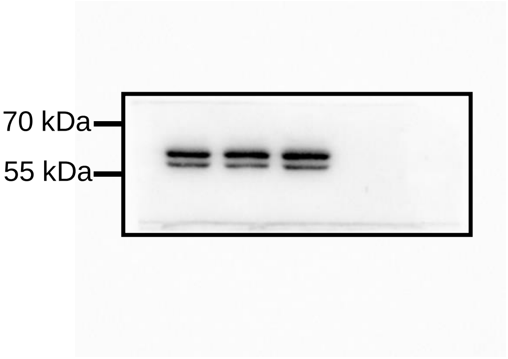

786-O AKT

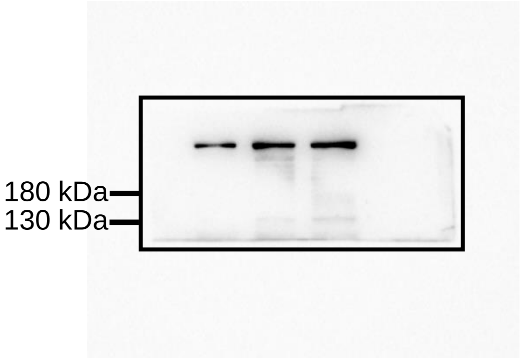

Ketr-3 p-mTOR

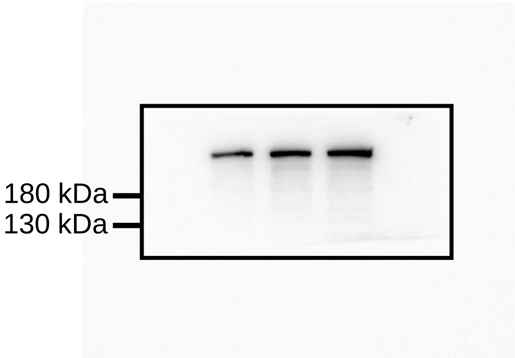

786-O p- mTOR

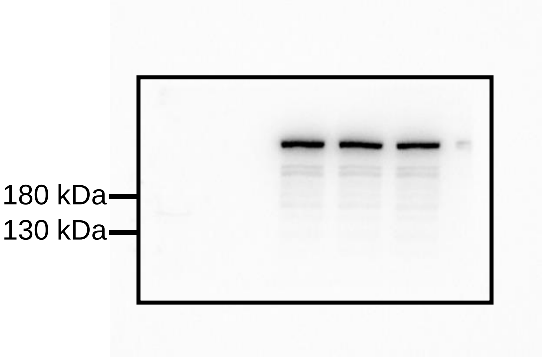

Ketr-3 mTOR

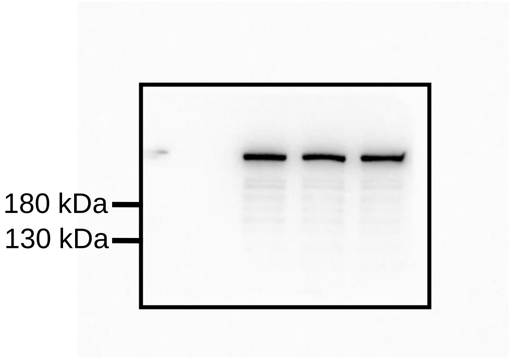

786-O mTOR

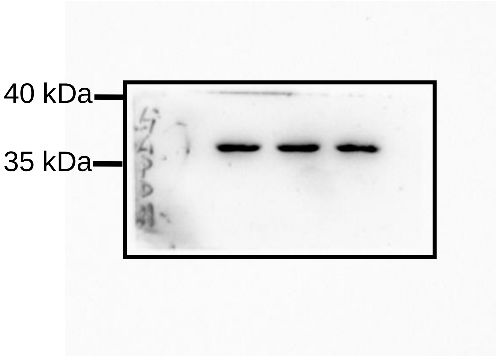

Ketr-3 GAPDH

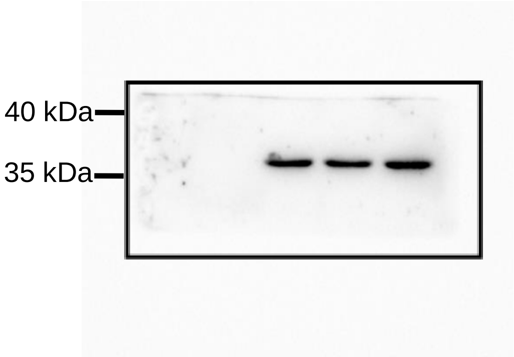

786-O GAPDH

B

The second replicate blots of figure 7B

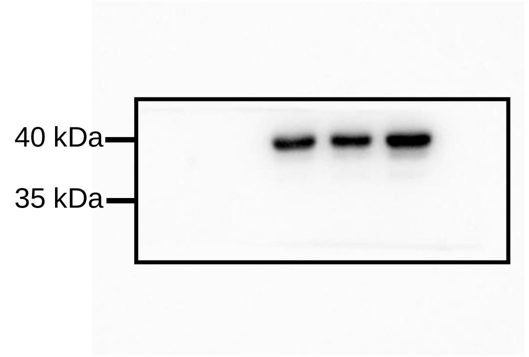

Ketr-3 AKT1S1

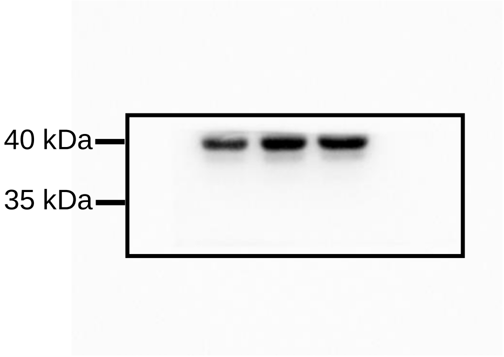

786-O AKT1S1

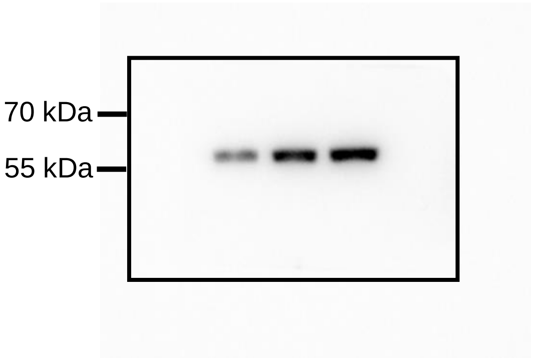

Ketr-3 p-AKT

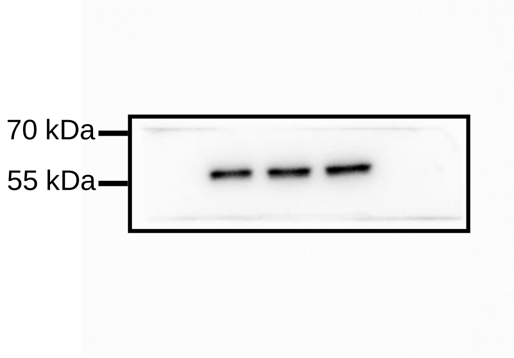

786-O p-AKT

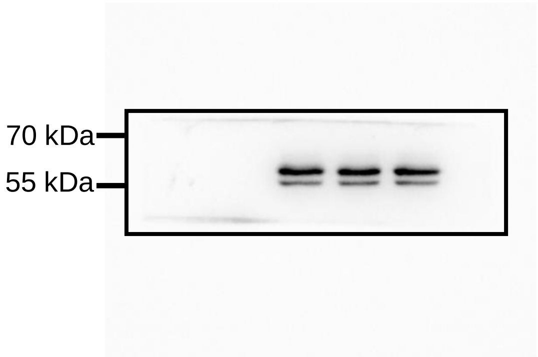

Ketr-3 AKT

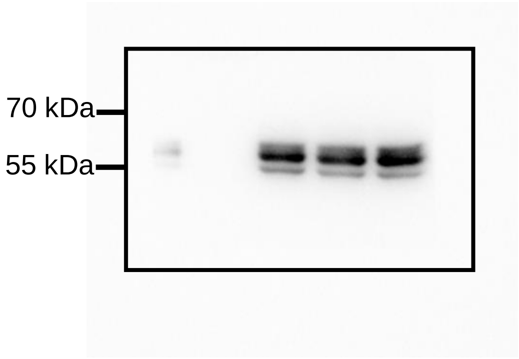

786-O AKT

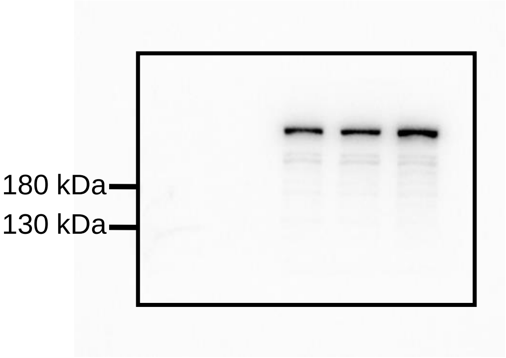

Ketr-3 p-mTOR

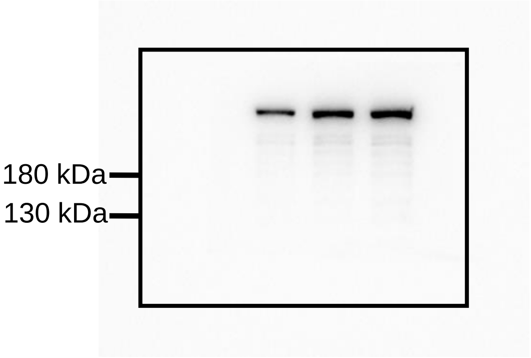

786-O p- mTOR

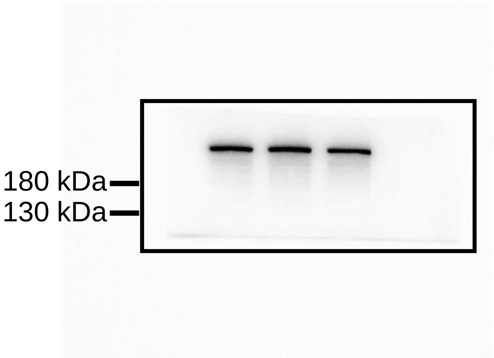

Ketr-3 mTOR

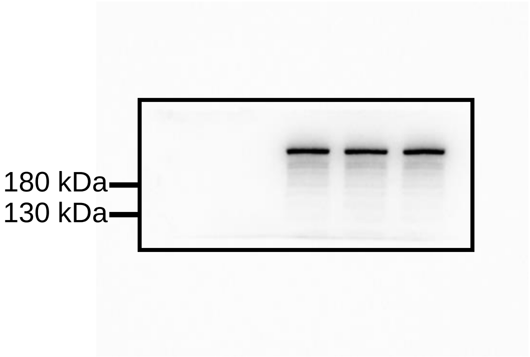

786-O mTOR

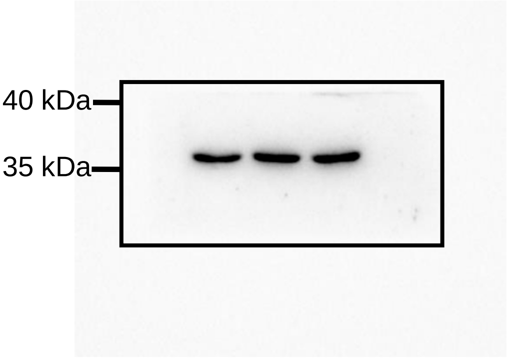

Ketr-3 GAPDH

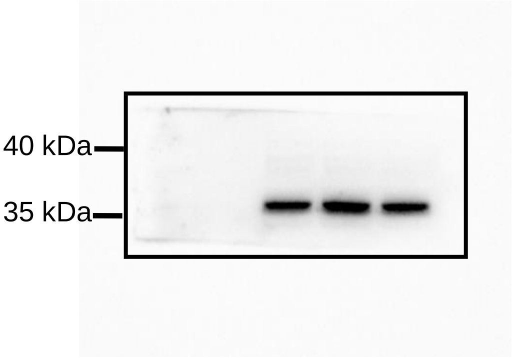

786-O GAPDH

C

The third replicate blots of figure 7B

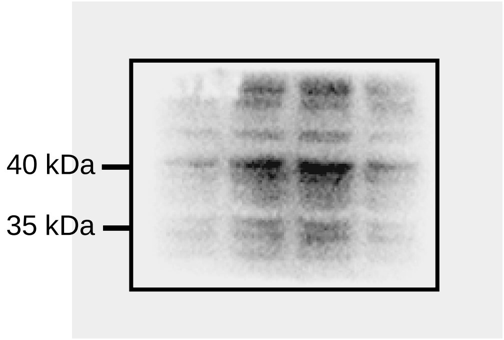

Ketr-3 AKT1S1

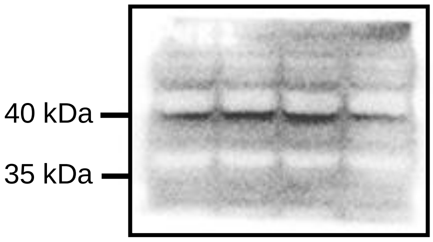

786-O AKT1S1

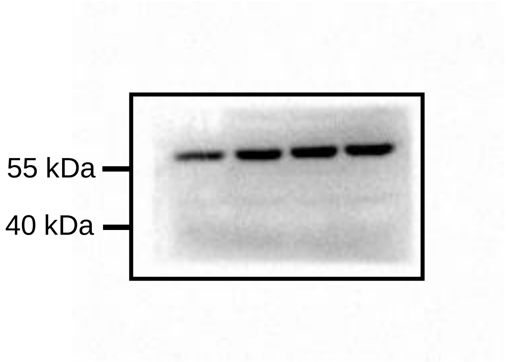

Ketr-3 p-AKT

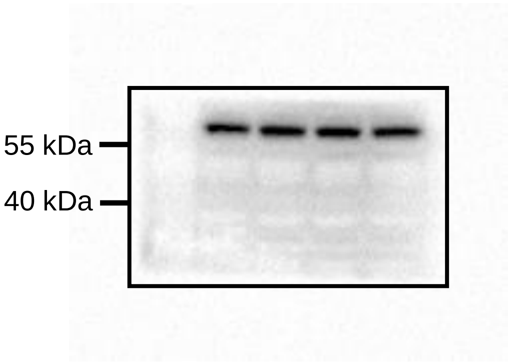

786-O p-AKT

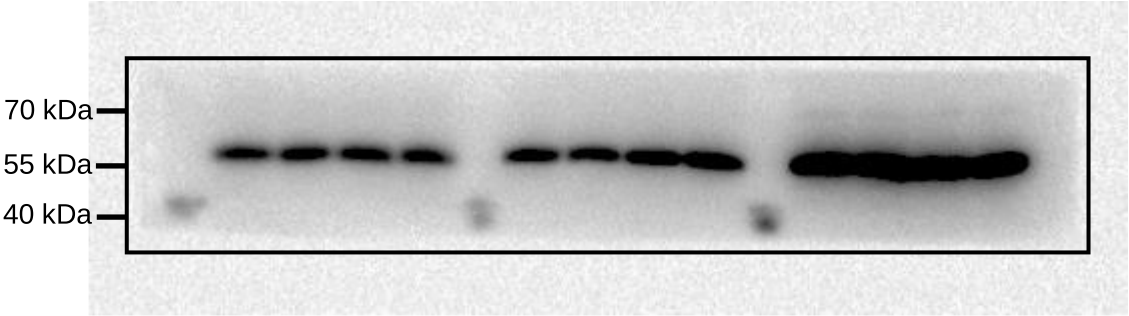

AKT

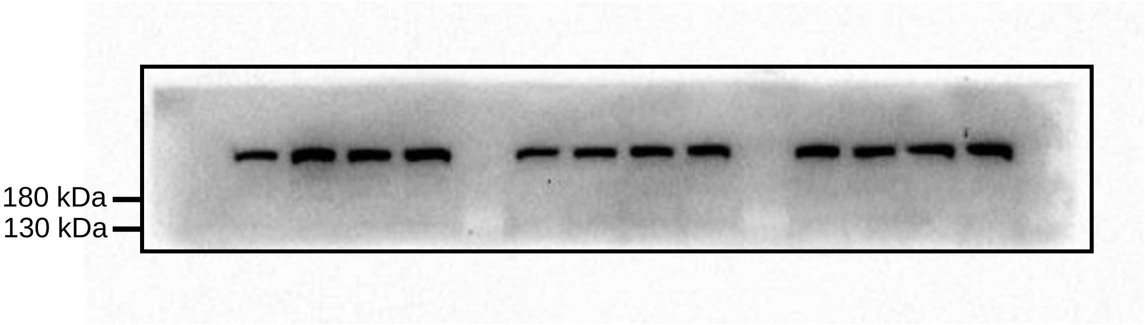

p-mTOR

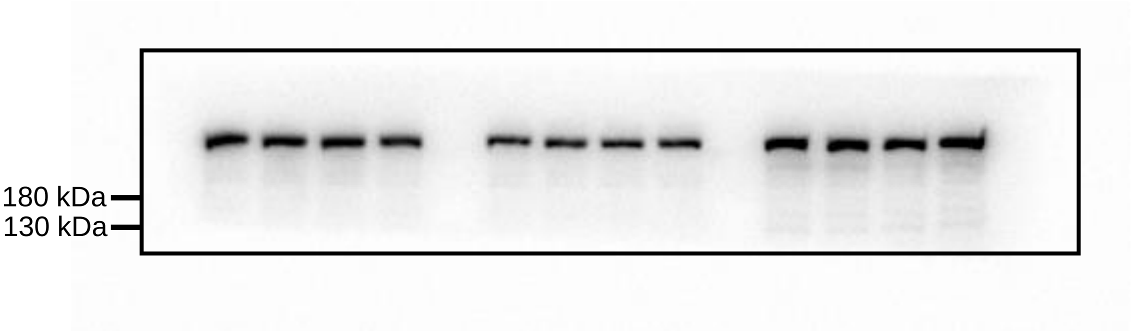

mTOR

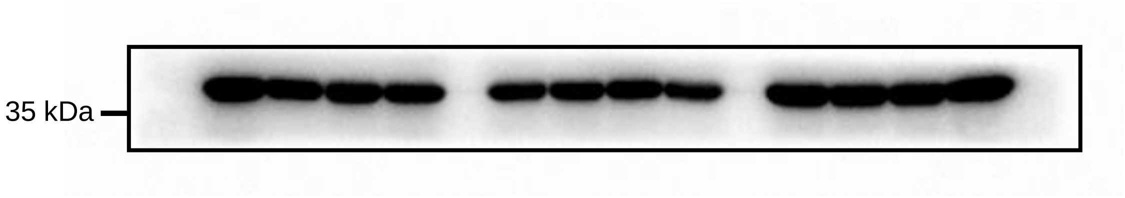

GAPDH
